# Vibration Isolation Properties of Novel Spacer Fabric with Silicone Inlay

**DOI:** 10.3390/polym15051089

**Published:** 2023-02-22

**Authors:** Annie Yu, Sachiko Sukigara, Arata Masuda

**Affiliations:** 1 Faculty of Fiber Science and Engineering, Kyoto Institute of Technology, Matsugasaki, Sakyo-ku, Kyoto 606-8585, Japan; 2 Mechanical Engineering Department, Kyoto Institute of Technology, Matsugasaki, Sakyo-ku, Kyoto 606-8585, Japan

**Keywords:** 3D knitted fabric, vibration transmissibility, damping, silicone tube, inlay, spacer structure

## Abstract

Spacer fabrics are good for impact force absorption and have the potential for vibration isolation. Inlay knitting of additional material to the spacer fabrics can give reinforcement to the structure. This study aims to investigate the vibration isolation properties of three-layer sandwich fabrics with silicone inlay. The effect of the presence of the inlay, inlay patterns and materials on the fabric geometry, vibration transmissibility and compression behaviour were evaluated. The results showed that the silicone inlay increases the unevenness of the fabric surface. The fabric using polyamide monofilament as the spacer yarn in the middle layer creates more internal resonance than that using polyester monofilament. Silicone hollow tubes inlay increases the magnitude of damping vibration isolation, whereas inlaid silicone foam tubes have the opposite effect. Spacer fabric with silicone hollow tubes inlaid by tuck stitches has not only high compression stiffness but also becomes dynamic, showing several resonance frequencies within the tested frequency range. The findings show the possibility of the silicone inlaid spacer fabric and provide a reference for developing vibration isolation materials with knitted structure and textiles materials.

## 1. Introduction

Long-term exposure of the human body to vibration can have harmful effects and bring negative impacts to the vascular, neurological and musculoskeletal systems. For instance, vibration produced by machinery is transmitted to the seat of the operator or vehicles to the feet of the driver, and then the exposure affects the entire body, which can cause lumbar spine and back problems [1,2,3]. In work-related tasks, there are many situations that expose the hands of workers to vibration, such as the use of electrical and pneumatic powered hand tools, which can include ballast machines, chain saws, straight grinders, high-speed dental drills, etc. [4,5]. Prolonged exposure of the hand to such vibrations could lead to numbness, pain, peripheral neuropathy, secondary Raynaud’s phenomenon and musculoskeletal problems of the hands and fingers [6,7,8,9]. Full neurological damage is irreversible and hand function is largely reduced.

In order to minimize vibration exposure, vibration isolation materials and anti-vibration gloves are often used. Conventional materials used as vibration isolators include elastomers, elastomeric foam, resilient gel, cork, air bladders, etc. The essential characteristic of vibration isolators is their stiffness which provides adequate load support and damping which governs the energy dissipating mechanism. The stiffness and damping functions can be provided by a single element or separate materials. Different kinds of materials can vary in stiffness, natural frequency characteristics and damping properties to impart different vibration isolation properties [10]. The current design of wearable anti-vibration products and gloves mainly focuses on the vibration isolation function when selecting materials. An increase in thickness and softness of the anti-vibration materials can help to improve their effectiveness in reducing the transmission of vibrations. However, when used as an anti-vibration glove, the vibration isolation materials would also increase difficulties in grasping and controlling hand tools, which incur safety risks. Therefore, it is valuable to have a material that can have good vibration isolation properties and, at the same time, can provide good wear comfort, thermal conductivity, air permeability, moisture transfer and bending softness.

Knitting is a method for producing a fabric (two-dimensional) by interloping of yarns (one-dimensional). Knitted fabrics always appear in daily life as clothing and home furnishings. A weft-knitted structure is elastic, while warp-knitting gives a stable fabric structure and needs to adopt elastic yarns to provide elasticity. Both weft and warp knitting methods allow the fabrics to fit the body contour well and facilitate body motions. A three-dimensional (3D) fabric called spacer fabric can also be produced by knitting. Spacer fabrics can be fabricated through both warp and weft knitting and are commonly used as cushioning materials. The fabric consists of three layers. The surface layers can provide a good hand feel, while the middle layer which connects the two surface layers can be made by using different types of filament yarns and connective patterns to provide different compression stiffnesses and behaviours. Having the characteristics of a knitted fabric, spacer fabrics are flexible and air-permeable which mean that they are suitable for protective garments or gloves. The connective layer of the fabric contains voids which not only substantially reduce the weight of the fabric but also provide a cushioning and damping effect on vibration. Weft-knitted spacer fabrics can provide negative stiffness within a certain range of compression displacement which could be effective for isolating vibration [11,12]. A number of studies have been carried out to understand the vibration isolation and damping properties of warp- and weft-knitted spacer fabrics. For instance, Blaga and Seghedin [13] and Blaga et al. [14] evaluated spacer fabrics under free vibration and showed that spacer fabrics have good capacity to absorb vibration energy. Chen et al. [15,16] found that fabric thickness and density, the diameter of filament yarns and the yarn arrangement of the connective layer significantly affect the vibration transmission and damping properties of spacer fabrics. Chen et al. [17] and Liu and Hu [18] observed that an increase in the thickness of spacer fabric, acceleration level and load mass lead to a decrease in the resonance frequency. Frydrysiak and Pawliczak [19] confirmed the vibration isolation capabilities of spacer fabric within a low frequency range of 1 to 100 Hz. When spacer fabrics are used as protective clothing or seat cushions, it is crucial but challenging to construct spacer fabric with low stiffness to provide greater vibration isolation yet at the same time has high loading support capacity to prevent the fabric structure from collapsing due to the stress imparted by the human body. Therefore, the application of spacer fabrics is still limited to cushioning purposes and is seldom used as the primary vibration isolation materials.

Inlay knitting is a method that applies extra yarns to modify the mechanical performance of a knitted fabric. Inlay yarns are not essential in making a knitted fabric but can provide additional reinforcement. In weft knitting, inlaid yarns can be incorporated into the knitted structure in the course direction as miss stitches with some tuck stitches at certain points of connection but never formed into a knitted loop. The inlay could affect the stability, elastic stretch and recovery, handle, weight, surface properties and visual appearance of a knitted structure [20]. The inlay knitting technique can be used to apply elastic yarns to the openings of knitted socks and gloves to provide stretchability for donning and holding in position. High-performance materials such as aramid, fibreglass, basalt, carbon, etc. have been used as inlaid yarns in the fabrication of composites to improve mechanical behaviour [21,22,23]. Previously, we developed a novel spacer fabric with silicone tubes inlaid into the connective layer [24,25]. The fabric has a significantly higher compression resistance and increased ability to absorb impact forces. It is meaningful to understand the effect of inlay on vibration transmissibility of spacer fabric.

In this study, silicone hollow tubes and silicone foam tubes are adopted as the inlay materials in a conventional spacer fabric structure to support the load, whereas thin (0.12 mm) monofilament yarns are used to reduce the fabric stiffness. The aim of this study is to investigate the effect of the inlay materials in the connective layer of spacer fabric on vibration isolation. The impacts of the presence of the inlay, and inlay patterns and materials are subsequently evaluated.

## 2. Materials and Methods

### 2.1. Fabric Samples

Five fabric samples were made by using a 10-gauge v-bed flat knitting machine (SWG091N210G, Shima Seiki, Wakayama, Japan). The samples were made of the same surface yarn, surface structure and spacer structure. A 450D 3-ply 100% polyester draw textured yarn together with a 140D 100% spandex yarn were used as the surface yarns. They were knitted into a single jersey structure for all of the samples. The samples were composed of two different spacer yarns and two inlay materials (Table 1).

Since spacer yarns connect the two knitted layers, the pattern and materials used directly affect the physical and mechanical properties of the resultant fabric. Polyamide and polyester monofilaments are commonly used as the spacer yarn. Four of the samples were fabricated with polyester monofilament yarn and 1 sample with polyamide monofilament. The dynamic stiffness of a material is related to its vibration isolation properties. Based on our previous study [26], spacer fabric that is thicker and spacer yarn that has a longer linking distance can provide a higher degree and range of vibration isolation. Therefore, all of the samples were constructed from monofilament yarns with a diameter of 0.12 mm, and a structure with spacer yarn that has a linking distance of 8 needles was adopted for all of the samples. The effect of spacer yarn materials can be evaluated.

In our previous study [24], inlay yarns were used for the middle connective layer of the spacer fabric to give extra support to the fabric structure. It is found that the density of inlay can slightly affect the compression properties. In preparing the inlaid samples, silicone hollow tubes and silicone foam tubes with a diameter of 1 mm were used as the inlay materials. The inlay yarn density was fixed and applied at every 4 courses on the fabric surface. Samples with two different inlay patterns, inlaying by using all miss stitches and inlaying by using both tuck and miss stitches, were produced for comparison. The details of the five fabric samples are shown in Table 2. The thickness of the samples was measured according to ISO 5084:1997-Textiles-Determination of thickness of textiles and textile products, under a pressure of 0.5 kPa with a circular plane intender of size 2 cm^2^. The course and wales densities of the fabrics were measured from the microscopic views at 10 different locations. All of the samples were placed in room temperature (20 ± 2 °C, 65 ± 2% relative humidity) to relax for 1 week after they were removed from the knitting machine.

### 2.2. Evaluation

#### 2.2.1. Vibration Transmissibility Evaluation

**Figure 1:**
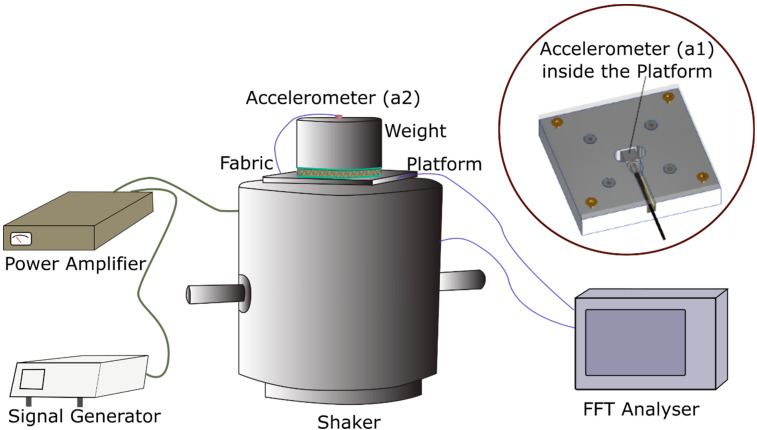
Set-up to evaluate vibration transmissibility of spacer fabrics.

The vibration isolation properties of the fabric samples were evaluated in accordance with ISO 13753—Method for measuring the vibration transmissibility of resilient materials when loaded by the hand-arm system. The samples were prepared in a circular shape with a diameter of 90 mm. The set-up for the vibration test included a vibration generator (513-B, EMIC Corporation, Tokyo, Japan), multifunction generator (WF1974, NF Corporation, Yokohama, Japan), power amplifier (371-A, EMIC Corporation, Japan), 2 accelerometers, a fast Fourier transform (FFT) analyser, platform and weight (Figure 1). A flat square platform made of stainless steel with dimensions of 100 × 100 × 21 mm was designed to place an accelerometer in the centre of the platform which was fixed onto the vibration generator. The fabric samples were placed on the platform and a cylindrical weight of 2.5 kg with a diameter of 90 mm was placed on top of the samples. The cylindrical weight is equivalent to that found when materials are gripped by the hand as specified in ISO 13753. Another accelerometer was placed on the top and in the centre of the weight. The vibration generator was excited by the sinusoidal signals controlled by a signal generator and transmitted through a power amplifier with an excitation magnitude of 1 m/s^2^. A sweeping cycle was performed between 20 Hz and 1000 Hz. A sweep test can identify the resonances of the samples. The nonlinear behaviour of the fabric can thus be analysed. The accelerations at the centre of the platform, a1, and the top of the mass, a2, measured by the two accelerometers were processed by using the FFT analyser. The vibration transmissibility *T* (dB) is expressed as $T=20\log_{10}|A_{2}/A_{1}|$, where *A*_1_ and *A*_2_ are the Fourier transforms of a1 and a2, respectively. The transmissibility represents the fraction of the applied excitation transmitted through the spacer fabric to the part being protected.

#### 2.2.2. Compression Properties Evaluation

A compression test on the fabric samples was carried out by using a universal testing machine (EZ-S, Shimadzu, Kyoto, Japan), which used upper and lower compression plates with a diameter of 118 mm. The testing specimens were prepared in the same circular shape and size as those used in the vibration test with a diameter of 9 cm. The compression speed was 12 mm/min with a maximum compression strain/stress of 500 N. Three specimens of each sample were tested.

## 3. Results and Discussion

### 3.1. Spacer Fabric Geometry

**Figure 2:**
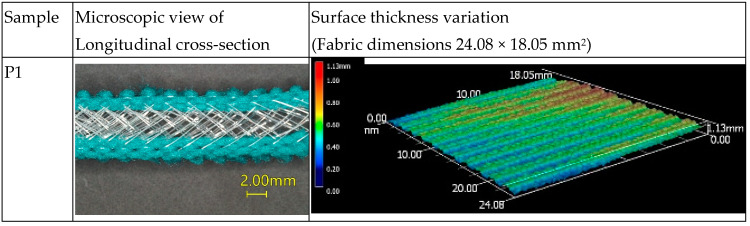

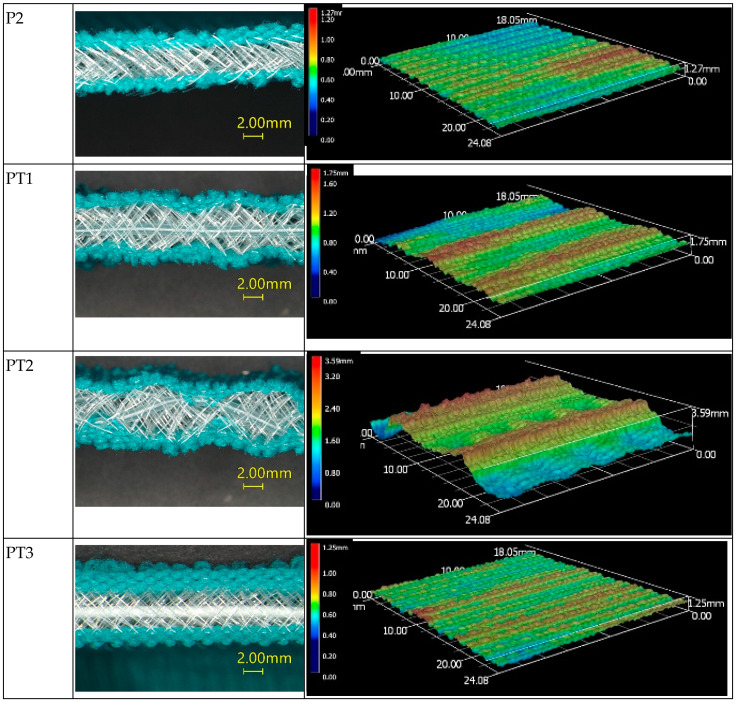
Spacer fabric samples geometry.

A 3D-optical microscope (VR-3000, KEYENCE, Osaka, Japan) was used to determine that the inlaying of silicone tubes into the connective layer of the spacer fabric changes the fabric geometry. The cross-sectional views and surface thickness variations of the samples are presented in Figure 2. From the longitudinal cross-section, the inlaid silicone tubes across the connective layer of the spacer fabric and its effect on the monofilament spacer yarn can be observed. The surface thickness variation can provide information about the surface evenness over an area of 24.08 × 18.05 mm^2^.

Spacer fabrics P1 and P2 have a similar fabric thickness and their surface is relatively flat compared with the fabrics that have an inlay. The presence of the inlay causes the fabric surface to become wavy. The inlaid tubes have a certain degree of elasticity which contracts the fabric structure, thus resulting in a higher wale density, greater thickness and a wavy fabric surface. Both PT1 and PT3 have inlaid tubes that run along the fabric course with all miss stitches. The silicone hollow tubes have a higher Young’s modulus than the silicone foam tubes and hence impart a higher degree of fabric shrinkage in PT1 along the course direction. Therefore, the surface of PT1 becomes wavy. In PT2, the inlaid silicone foam tubes alternatively form tuck stitches with the front and back needle beds. Therefore, the variation of the surface thickness is the highest amongst the three types of inlaid spacer fabrics. The variations of the fabric geometry caused by the inlay not only affect the appearance of the fabrics but also can have an impact on the vibration transmissibility and compression properties of the spacer fabrics.

### 3.2. Compression Properties

**Figure 3:**
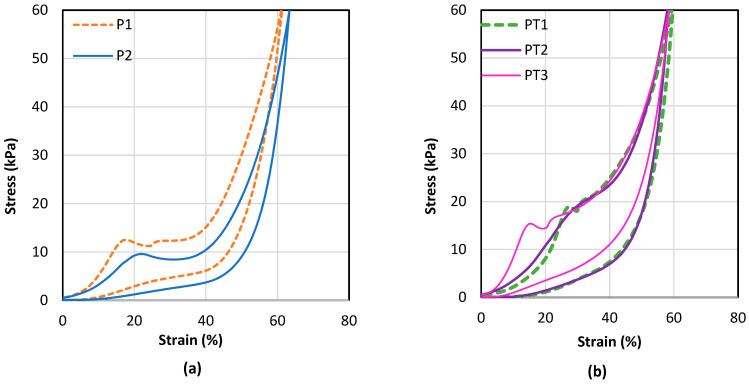

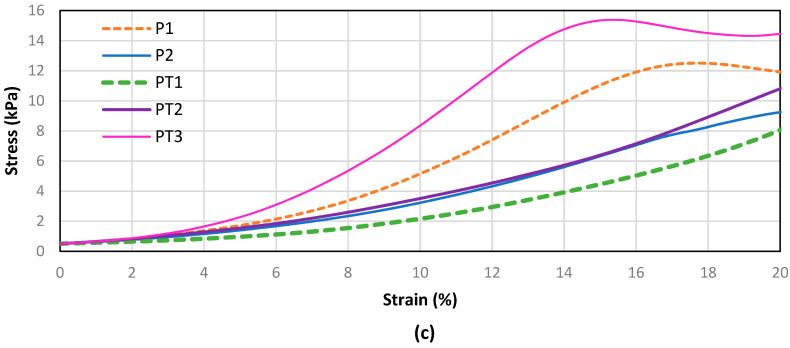
Compression stress-strain curves of (**a**) spacer fabric samples without inlay, (**b**) spacer fabric samples inlaid with silicone-based materials and (**c**) all of the fabric samples with a load strain up to 20%.

The compression properties of the spacer fabric samples are non-linear as shown in Figure 3. Spacer fabric P1 is constructed of polyester monofilaments which have a higher Young’s modulus than polyamide monofilaments. Spacer fabric P1 requires a higher stress than P2 to be compressed to the same strain. This is in agreement with Yu et al. [24] that spacer fabric with polyester spacer yarn has a higher compression stiffness than that with polyamide yarn. The slopes of the stress-strain curves of both P1 and P2 start to decrease and become negative at a stress of 12 kPa and 9 kPa, respectively. The negative stiffness effect could be due to the shearing, buckling and rotating of the spacer yarns. The negative stiffness effect of PT1 and PT3 appear at a higher stress than P1 and P2. This confirms that the presence of an inlay along the connective layer can reinforce the spacer structure to withstand a higher stress. Spacer fabric PT2 consists of silicone inlaid tubes that alternatively form the front and back tuck stitches with the two surface layers, thus resulting in a more complex connective structure. This structure imparts a higher compression stiffness to PT2 in comparison to PT1 during compression up to a strain of 20% and prevents negative stiffness during compression.

Figure 3c shows the initial behaviour of the spacer fabric samples with a compression strain up to 20%. The slopes of the stress-strain curves are relatively smaller at the beginning and gradually increase and tend to be linear when the stress increases. The Young’s modulus is regarded as the ratio of engineering stress to engineering strain which is the slope of the stress-strain curve in the linear elastic region. Spacer fabric P1 has a higher Young’s modulus than P2. It is interesting to see that PT1 and PT2 have a lower Young’s modulus than P1 during compression up to strain of 20%. This can be explained by the wavy surface caused by the inlaid silicone tubes. In the initial compression, only the convex parts come into contact with the indenter and support against the compression force. A wavy surface is less supportive than a flat surface. When the compression stress is increased, the fabric tends to flatten and becomes more compact and stiffer against the compression. On the other hand, PT3 has the highest Young’s modulus during the initial compression. The relatively flat surface of PT3 and the inlaid silicone foam tubes significantly increase the compression strength of the spacer fabric.

### 3.3. Vibration Transmissibility

**Figure 4:**
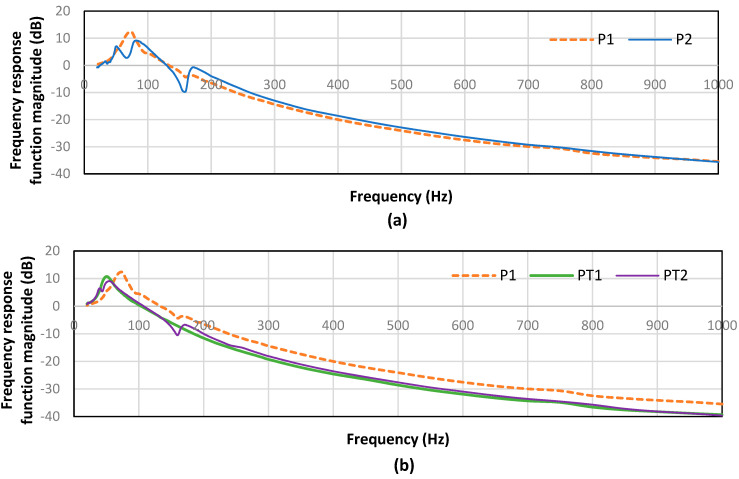

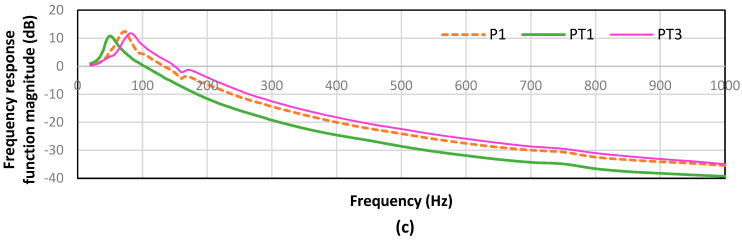
Vibration transmissibility of fabric samples that show the effect of: (**a**) spacer yarn, (**b**) inlay pattern and (**c**) inlay materials.

The vibration transmissibility of the spacer fabric samples is compared and presented in Figure 4. When the magnitude of the frequency response function (FRF) is greater than 0 dB, the vibration is amplified and vice versa. Figure 4 shows that all of the fabric samples can lead to a notable vibration reduction in certain frequency ranges. However, the resonant frequency, magnitude of the resonance and frequency range of the vibration reduction differ for the different types of fabric samples. In the vibration experiment, the spacer fabric acts as a vibration damper. The effect of the spacer yarn, inlaid method and inlaid materials on vibration isolation are subsequently discussed.

#### 3.3.1. Effect of Spacer Yarn

Spacer fabrics P1 and P2 are conventional spacer fabrics with the same structure and type of surface yarns and have a similar fabric thickness, but use different types of spacer yarns. As observed in Figure 4a, P1 and P2 have similar vibration damped frequency bands of 133–1000 Hz and 129–1000 Hz, respectively. However, P1 shows a natural frequency at 75 Hz which has a higher amplitude than the natural frequency of P2 and appears at 81 Hz. Apart from the resonance peak at the natural frequency, two more peaks with valleys can be observed on the FRF curve of P2 at 51 Hz and 170 Hz. A small peak is also found at 170 Hz in the FRF curve of P1. This suggests the internal resonance of the spacer fabrics. Using polyamide monofilaments as the spacer yarn can increase the internal resonance toward vibration. The magnitude of the resonance of P2 at 170 Hz is higher than that of P1. Polyamide monofilament yarns have a lower Young’s modulus than polyester monofilament yarns which result in a softer spacer fabric and a lower compression stiffness and allow for dynamic vibration transmission within the spacer structure. Due to the presence of resonance, P2 can better isolate vibration at around 160 Hz as opposed to P1. On the other hand, P1 can isolate vibration slightly better than P2 at a frequency range of 180 Hz to 300 Hz. At a frequency range from 300 Hz to 1000 Hz, the vibration isolation ability of P1 and P2 becomes similar.

#### 3.3.2. Effect of Inlay Pattern

Spacer fabrics PT1 and PT2 are inlaid with silicone hollow tubes, with a natural frequency of 50 Hz and 54 Hz, respectively. Spacer fabric PT1 started to isolate vibration at an excitation frequency of 104 Hz and PT2 at 107 Hz. In comparison to P1, PT1 and PT2 showed a reduction in their natural frequency and a wider damped frequency range and higher magnitude of vibration isolation (Figure 4b). The natural frequency of PT1 and PT2 also shows a lower peak which means a higher magnitude of damping. This shows that inlaying silicone hollow tubes into the spacer fabric can improve the ability of the fabric to isolate vibration. The inlay increases the thickness of the fabrics and reduces the initial stiffness, both of which facilitate vibration isolation.

The inlay pattern of the silicone tubes in PT1 and PT2 differs. Within the tested frequency range, the FRF of PT1 shows only one peak for its natural frequency whereas three peaks can be observed for the FRF of PT2. With the exception of the excitation frequency near resonance, the FRFs of PT1 and PT2 are similar. The silicone tube is inlaid into the connective layer by using all miss stitches for PT1 and miss and tuck stitches for PT2. The tuck stitches of the inlaid tubes increase the number of resonances within the spacer structure. The tucking of the silicone hollow tubes not only changes the surface geometry but also the vibration transmissibility of the spacer fabric.

#### 3.3.3. Effect of Inlay Materials

The knitting and inlay patterns of PT1 and PT3 are the same but the vibration transmissibility of the two fabrics is very different. Silicone foam materials are a porous viscoelastic polymer that is foamed from silicone rubber and commonly used as vibration isolation materials [27,28]. However, the inlaid silicone foam tubes in PT3 not only fail to improve the vibration isolation ability of the spacer fabric, but also impart a higher natural frequency to PT3 and a narrower band of vibration isolation than P1 (Figure 4c). The magnitude of the FRF is even higher than that of the spacer fabric without inlay for the frequency band of 80–1000 Hz. Therefore, the vibration isolation ability of the spacer fabric is increased with the inlaying of silicone hollow tubes (PT1), but reduced with the inlaying of silicone foam tubes (PT3). Silicone foam tubes with a relatively lower Young’s modulus may not be suitable to use as inlay for spacer fabric to provide vibration isolation.

The inlaid tubes have different elastic properties which can vary the thickness, surface geometry and compression properties of the spacer fabric and thus the variations in vibration isolation ability. As such, a thicker fabric with a lower initial compression stiffness could better contribute to isolating vibration.

## 4. Conclusions

Spacer fabrics are a potentially good material for items that isolate vibration, such as work gloves, insoles or protective garments. Inlays of silicone-based materials into the connective structure of spacer fabrics can enhance their compression stiffness and allow the fabrics to better absorb the impact forces. In this study, the idea of using inlays of silicone tubes into the spacer fabrics to form a specific sandwich structure for improving the vibration transmissibility was investigated. The geometry, compression properties and vibration isolation performance of spacer fabrics with and without inlays have been evaluated. It is important to understand the impact of inlays on the vibration transmissibility of spacer fabrics. The effects of the type of spacer yarn, inlay pattern and inlay materials were analysed. The following conclusions are made based on the experimental results:

1. The presence of silicone inlay can affect the surface appearance and evenness and the geometry of the connective layer. Hence, this affects the compression behavior of the spacer fabric.
2. The use of polyamide monofilaments as the spacer yarn increases the internal resonance of the spacer fabric with excitation frequency. The difference between spacer fabrics made of polyamide and polyester monofilaments in the vibration isolation ability is small at a frequency range from 300 Hz to 1000 Hz.
3. Spacer fabric inlaid with silicone hollow tubes can increase the magnitude of damping vibration isolation, whereas inlaid silicone foam tubes with a relative lower Young’s modulus have the opposite effect.
4. When the silicone hollow tubes are inlaid into the spacer fabric with tuck stitches, the fabric structure has a higher compression stiffness and becomes dynamic, showing several resonance frequencies within the tested frequency range.

The findings of this study can act as a reference for developing a knitted material for vibration isolation that aims to give better wearing comfort. Although only five fabrics samples with the same spacer yarn connection are produced and evaluated, the results reveal that inlaying silicone hollow tubes into spacer fabric can improve the vibration isolation ability of the fabric so that the fabric can be potentially used as material for anti-vibration gloves or garments. A systematic evaluation that involves a larger number of samples that take some other factors, such as the spacer structure, inlay yarn density, etc., into account is recommended for future studies.

## Figures and Tables

**Table 1:** Details of spacer yarn and inlay yarn used to construct fabric samples.

	Content	Thickness (mm)	Young’s Modulus
Spacer yarn	Polyamide monofilament	0.12	1.66 GPa
	Polyester monofilament	0.12	3.33 GPa
Inlay yarn	Silicone hollow tube	1	0.816 MPa
	Silicone foam tube	1	0.263 MPa

**Table 2:** Details of the fabric samples.

Sample No.	Monofilament Spacer Yarn	Inlaid Yarn & Pattern	Fabric Weight (g/m^2^)		Thickness (mm)		Course Density (Course/cm)		Wale Density (Wale/cm)	
P1	PET 0.12 mm	No inlay	743.8	±5.0	6.1	±0.2	18.9	±0.2	4.7	±0.0
P2	PA 0.12 mm	No inlay	784.0	±6.3	6.4	±0.1	17.3	±0.1	4.9	±0.1
PT1	PET 0.12 mm	Silicone hollow tube, All miss stitches	1247.3	±38.8	8.4	±0.3	16.8	±0.2	5.5	±0.1
PT2	PET 0.12 mm	Silicone hollow tube, 1 front tuck 5 miss 1 back tuck 5 miss	1273.3	±49.6	8.5	±0.4	18.4	±0.5	5.8	±0.7
PT3	PET 0.12 mm	Foam tube, All miss stitches	1027.8	±30.5	7.1	±0.1	17.0	±0.2	5.2	±0.0

## Data Availability

Not applicable.

## References

[1] Okunribido O.O., Shimbles S.J., Magnusson M., Pope M. (2007). City bus driving and low back pain: A study of the exposures to posture demands, manual materials handling and whole-body vibration. Appl. Ergon..

[2] Degan G.A., Coltrinari G., Lippiello D., Pinzari M. (2018). Effects of ground conditions on whole-body vibration exposure on cars: A case study of drivers of armored vehicles. WIT Trans. Built Environ..

[3] Dong R.C., Guo L.X. (2017). Effect of muscle soft tissue on biomechanics of lumbar spine under whole body vibration. Int. J. Precis. Eng. Manuf..

[4] Barregard L., Ehrenström L., Marcus K. (2003). Hand-arm vibration syndrome in Swedish car mechanics. Occup. Environ. Med..

[5] Vihlborg P., Bryngelsson I.-L., Lindgren B., Gunnarsson L.G., Graff P. (2017). Association between vibration exposure and hand-arm vibration symptoms in a Swedish mechanical industry. Int. J. Ind. Ergon..

[6] Chetter I.C., Kent P.J., Kester R.C. (1997). The hand arm vibration syndrome: A review. Cardiovasc. Surg..

[7] Brammer A.J., Taylor W., Lundborg G. (1987). Sensorineural stages of the hand-arm vibration syndrome. Scand. J. Work Environ. Health.

[8] Gemne G. (1997). Diagnostics of hand-arm system disorders in workers who use vibrating tools. Occup. Environ. Med..

[9] Gerhardsson L., Hagberg M. (2014). Work ability in vibration-exposed workers. Occup. Med..

[10] Ungar E.E. (2007). Damping of Structures and Use of Damping Materials. Handbook of Noise and Vibration Control.

[11] Chen F., Hu H., Liu Y. (2015). Development of weft-knitted spacer fabrics with negative stiffness effect in a special range of compression displacement. Text. Res. J..

[12] Yu A., Sukigara S., Takeuchi S. (2020). Effect of inlaid elastic yarns and inlay pattern on physical properties and compression behaviour of weft-knitted spacer fabric. J. Ind. Text..

[13] Blaga M., Seghedin N. (2017). Knitted spacer fabrics behaviour at vibrations. J. Text. Eng. Fash. Technol..

[14] Blaga M., Harpa R., Seghedin N.E., Marmarali A., Ertekin G. (2018). Evaluation of the knitted fabrics stiffness through dynamic testing. IOP Conf. Ser. Mater. Sci. Eng..

[15] Chen C., Chen J., Sun F., Yang H., Du Z. (2018). Analysis of the damping property of warp-knitted spacer fabrics under damped free vibration. Text. Res. J..

[16] Chen C., Chen J., Sun F., Yang H., Lv Z., Zhou Q., Du Z., Yu W. (2018). Study of the vibration transmission property of warp-knitted spacer fabrics under forced sinusoidal excitation vibration. Text. Res. J..

[17] Chen F., Liu Y., Hu H. (2016). An experimental study on vibration isolation performance of weft-knitted spacer fabrics. Text. Res. J..

[18] Liu Y., Hu H. (2015). Vibration isolation behaviour of 3D polymeric knitted spacer fabrics under harmonic vibration testing conditions. Polym. Test..

[19] Frydrysiak M., Pawliczak Z. (2021). Vibro-insulation properties for spacer knitted fabric as a comparative study. J. Ind. Text..

[20] Spencer D.J. (2001). 6—Comparison of weft and warp knitting. Knitting Technology.

[21] Balea L., Dusserre G., Bernhart G. (2014). Mechanical behaviour of plain-knit reinforced injected composites: Effect of inlay yarns and fibre type. Compos. Part B Eng..

[22] Dusserre G., Balea L., Bernhart G. (2014). Elastic properties prediction of a knitted composite with inlaid yarns subjected to stretching: A coupled semi-analytical model. Compos. Part A Appl. Sci. Manuf..

[23] Cheng K.B., Lee K.C., Ueng T.H., Mou K.J. (2002). Electrical and impact properties of the hybrid knitted inlaid fabric reinforced polypropylene composites. Compos. Part A Appl. Sci. Manuf..

[24] Yu A., Sukigara S., Yick K.L., Li P.L. (2020). Novel weft-knitted spacer structure with silicone tube inlay for enhancing mechanical behavior. Mech. Adv. Mater. Struct..

[25] Yu A., Sukigara S., Shirakihara M. (2021). Effect of Silicone Inlaid Materials on Reinforcing Compressive Strength of Weft-Knitted Spacer Fabric for Cushioning Applications. Polymers.

[26] Yu A., Sukigara S., Masuda A. (2020). Investigation of vibration isolation behaviour of spacer Fabrics with elastic Inlay. J. Text. Eng..

[27] Landrock A.H. (1995). Handbook of Plastic Foams: Types, Properties, Manufacture and Applications.

[28] Yan S., Jia D., Yu Y., Wang L., Qiu Y., Wan Q. (2020). Influence of γ-irradiation on mechanical behaviors of poly methyl-vinyl silicone rubber foams at different temperatures. Mech. Mater..

